# Substrate Specificity of Acyltransferase Domains for Efficient Transfer of Acyl Groups

**DOI:** 10.3389/fmicb.2018.01840

**Published:** 2018-08-07

**Authors:** Jie-Jie Shen, Fu Chen, Xiao-Xuan Wang, Xiao-Fang Liu, Xin-Ai Chen, Xu-Ming Mao, Yong-Quan Li

**Affiliations:** ^1^Institute of Pharmaceutical Biotechnology, Zhejiang University, Hangzhou, China; ^2^College of Life and Environmental Sciences, Shanghai Normal University, Shanghai, China; ^3^Key Laboratory of Microbial Biochemistry and Metabolism Engineering of Zhejiang Province, Hangzhou, China

**Keywords:** AT, allmal-CoA, ethmal-CoA, *self*- and *trans*-acylation, substrate specificity

## Abstract

Acyltransferase domains (ATs) of polyketide synthases (PKSs) are critical for loading of acyl groups on acyl carrier protein domains (A) via *self*- and *trans*-acylation reactions, to produce structurally diverse polyketides. However, the interaction specificity between ATs and unusual acyl units is rarely documented. In *Streptomyces* *tsukubaensis* YN06, we found that AT4_FkbB_ [an AT in the fourth module of tacrolimus (FK506) PKS] transferred both allylmalonyl (allmal) and emthylmalonyl (ethmal) units to ACPs, which was supposed responsible for the production of both FK506 and its analog FK520, respectively. Mutations of five residues in AT4_FkbB_ (Q119A, L185I-V186D-V187T, and F203L) caused decreased efficiency of allmal transfer, but a higher ratio of ethmal transfer, supposedly due to less nucleophilic attacks between Ser599 in the active site of AT4_FkbB_ and the carbonyl carbon in the allmal unit, as observed from molecular dynamics simulations. Furthermore, reverse mutations of these five residues in ethmal-specific ATs to the corresponding residues of AT4_FkbB_ increased its binding affinity to allmal-CoA. Among these residues, Val187 of AT4_FkbB_ mainly contributed to allmal recognition, and V187K mutant produced less FK520 than wild type. Our findings thus suggested that five critical residues within AT4_FkbB_ were important for AT functionality in polyketide extension and potentially for targeting biosynthesis by generating desirable products and eliminating undesirable analogs.

## Introduction

Polyketide natural products function as a wide range of therapeutic agents, such as immunosuppressants (Mo et al., 2011), anti-cancer agents (Dunn et al., 2013), antibiotics (Urem et al., 2016), which are invaluable natural resources for pharmaceutical development (Fegan et al., 2010; Dunn et al., 2013; Mo and Suh, 2016). Many polyketides are produced by type I assembly line PKSs containing several catalytic modules, each of which is responsible for one round of polyketide chain elongation. The essential catalytic module is basically comprised of β-ketoacyl synthase domain (KS), AT, and ACP domain (Fischbach and Walsh, 2006; Khosla et al., 2007; Fegan et al., 2010; Dunn et al., 2013). The KS within each module accepts a polyketide chain from the upstream ACP and condenses acyl units bound to KS and ACP. AT selects the appropriate acyl unit and load it onto the ACP via two-step reactions: a *self*-acylation reaction to recognize acyl units from acyl donors to the active site Ser to form acyl-*O*-AT intermediates; and a *trans*-acylation reaction to transfer the acyl units from AT intermediates to the phosphopantetheine arm of ACP to form acyl-*S*-ACPs (Khosla et al., 2007; Dunn et al., 2013; Ye et al., 2014).

Loading several different acyl units to produce structurally diverse polyketide backbones depends on natural ATs in modular PKSs specifically (Musiol and Weber, 2012; Dunn et al., 2013). Studies on ATs mainly focus on understanding the catalytic mechanism and substrate specificity at present. The catalytic processes of several ATs have been interpreted for their appropriate transfer of acyl groups (Marsden et al., 1994; Dunn et al., 2013; Musiol et al., 2013; Jiang et al., 2015; Wang Y.Y. et al., 2015). AT3 in 6-Deoxyerythronolide B Synthase (DEBS) recognizes only the electrophilic malonyl (M) component but not methylmalonyl (MM) component, which is strictly specific in the *self*-acylation reaction (Dunn et al., 2013). The discrete AT KirCI of kirromycin PKS transfers only M not MM or ethylmalonyl (ethmal) unit to ACP, which is specific in the *tran*-acylation reaction (Musiol et al., 2013). Structural studies of ATs have elucidated the catalytic mechanism and the key residues contributing to substrate specificity (Wong et al., 2011; Liew et al., 2012; Sundermann et al., 2013; Wang F. et al., 2015). Co-crystallization of AT_DY N10_ with acyl groups showed that AT_DY N10_ protects the M-enzyme intermediate to specifically facilitate the transfer of M to ACP and the bulky residues Phe752 and Met680 are primarily specific for M binding (Liew et al., 2012). Gln198 of AT6 in DEBS promotes the activation of the thioester group and helps to keep MM-CoA in a conformation suitable for the nucleophilic attack from the active site Ser197 (Sundermann et al., 2013). However, the mechanism of substrate specificity between ATs and acyl units still remains unclear.

FK506 and its analog FK520 as secondary metabolites are co-produced by *Streptomyces* *tsukubaensis* (Chen et al., 2012; Huang et al., 2013). In our industrial strain *S.* *tsukubaensis* YN06, the yield of FK506 and FK520 in shake-flask fermentation is 103.3 and 17.0 mg/L respectively, showing a ratio of FK506 : FK520 of 6.1:1 (**Figure 4F**). This process requires the transfer of a unique allylmalonyl (allmal) unit, synthesized by TcsA-D encoded within FK506 gene cluster (Goranovič et al., 2010; Mo et al., 2011; Barreiro and Martínez-Castro, 2014), and ethmal unit by AT4_FkbB_ (AT in the fourth module of FK506 PKS) (Jiang et al., 2015). So here we used AT4_FkbB_ as a model to study the molecular basis for substrate specificity of acyl units. We found AT4_FkbB_ transfers both allmal- and ethmal-CoA onto diverse ACPs in *self*- and *trans*-acylation reactions simultaneously. Five residues were identified to be potentially important for AT4_FkbB_ in allmal group recognition by protein alignment between AT4_FkbB_ and ethmal-specific ATs. The essentiality of these key AAs of AT4_FkbB_ were tested by mutations and it was found that those five AAs controlled allmal specificity. This information allowed us to enhance the transferred efficiency of allmal by AT4_FkbB_ via a single AA mutation. Our study thus provides new insights into the molecular basis of substrate specificity of ATs, and will contribute to targeting biosynthesis of structure-specific polyketides.

## Materials and Methods

### Production and Purification of Flag-Tagged ACP4_FkbB_ and AT4_FkbB_ of FK506 PKS, AT4_FkbB_ of FK520 PKS, AT4_TiA2_ of Fidaxomicin PKS, AT3_lsd12_ of Lasalocid PKS, AT5_monAIV_ of Monensin PKS and Their Mutants

The primers in this study were listed in **Supplementary Table S2**. The expression vectors of ACP4_FkbB_ and AT4_FkbB_ of FK506 PKS were constructed according to Jiang et al. (2015). The expression vectors of *AT4*_FkbB_ of FK520 PKS in *Streptomyces hygroscopicus* var. *ascomyceticus* (ATCC 14891) (Wu et al., 2000), AT3_lsd12_ of lasalocid PKS and *AT5*_monAIV_ of monensin PKS were synthesized by GenScript (Nanjing, China). *AT4*_TiA2_ of fidaxomicin PKS was amplified from the genomic DNA of *Actinoplanes deccanensis* ATCC21983 using primers P41 and P42 by PCR, while the strain grew at 30°C in ISP4 solid medium and TSB liquid medium (BD Biosciences, Shanghai, China). *AT4*_TiA2_ and Flag-tagged ACP4_FkbB_ were cloned into the expression vector pET28a (Novagen, Beijing, China) as *Nde*I and *Hin*dIII fragments, and confirmed by sequencing, resulting in the expression vector *AT4*_TiA2_- and *ACP4*_FkbB_-pET28. The expression vectors of mutants were amplified from the plasmids pET28a-*AT*s using primers P3-P40 and P43-P60 by PCR using a QuikChang site-directed mutagenesis kit (Strata-gene, Santa Clara, CA, United States), and confirmed by DNA sequencing. *E. coli* strain BL21 (DE3) containing the above expression vectors was grown in 200 ml of LB liquid medium (Oxoid, Beijing, China) at 37°C to OD_600_ = 0.4. Subsequently, the culture was added with IPTG (Sigma, Beijing, China) to a final concentration of 0.1 mM for induction overnight at 16°C. Cells were harvested by centrifugation (6000 rpm, 5 min). The cell pellets were resuspended in lysis buffer (20 mM Tris-HCl pH 8.0, 250 mM NaCl), and lysed by sonication (2 s on and 5 s off in total 20 min on ice). After centrifugation at 14000 rpm for 20 min, the supernatants were incubated with Ni-NTA agarose (Qiagen, Valencia, CA, United States) for 1 h. The resins were washed with 10 column volumes of lysis buffer, and the bound proteins were eluted with two column volumes of elution buffer (20 mM Tris-HCl pH 8.0, 250 mM NaCl, 250 mM imidazole). The elution buffer containing the protein was exchanged to dialysate buffer (20 mM Tris-HCl pH 8.0, 25 mM NaCl, 10% glycerol, 1 mM DTT) by dialysis bags in a beaker on ice and the dialysate buffer was changed every 3 h for three times. The proteins collected from dialysis bag were used for biochemical assays and cross-linking experiments.

### Biochemical Assays of Acyl Transfer

For biochemical analyses of *self*-acylation reactions of ATs, a reaction mixture in 50 μl contains 20 μM ATs, 200 μM allmal-CoA [ethmal-CoA or allmal-CoA:ethmal-CoA = 1:1. allmal-CoA and ethmal-CoA were chemically synthesized (Jiang et al., 2015)] and 100 mM Tris-HCl, pH 8.0. The reaction mixtures were incubated at 25°C for 1 h. ATs include AT4_FkbB_ from FK506 and FK520 PKS, AT4_TiA2_ from fidaxomicin PKS, AT3_lsd12_ from lasalocid PKS, AT5_monAIV_ from monensin PKS and their mutants. However, only the concentration of allmal- or ethmal-CoA was changed to 2 mM in the reactions of AT4_TiA2_ and its mutants in fidaxomicin PKS. Allylmalonylation and ethylmalonylation conversion yields in *self*-acylation reactions, comparing ion peak heights of acylated and intact protein in MS [allmal-AT/(allmal-AT+AT)^∗^100%], were selected the same to the retention time of HPLC (Jiang et al., 2015; Wang Y.Y. et al., 2015).

For biochemical synthesis of holo-ACPs, a mixture in 50 μl contains 16 μM apo-ACPs, 160 μM CoA, 2 μM phosphopantetheinyl transferase Sfp (Quadri et al., 1998), 1.25 mM MgCl_2_ and 100 mM Tris-HCl, pH 8.0, was incubated at 25°C for 0.5 h. Apo-ACPs include apo-ACP2/4_FkbB_ and ACP10_FkbA_, which were prepared as described previously (Wang Y.Y. et al., 2015).

For biochemical analyses of *trans*-acylation reactions, a reaction mixture in 50 μl contains 1.6 μM ATs, 16 μM allmal-CoA (ethmal-CoA or allmal-CoA:ethmal-CoA = 1:1, M-CoA, MM-CoA), 16 μM holo-ACPs obtained above and 100 mM Tris-HCl, pH 8.0. The reaction mixture was incubated at 25°C for 2 h. ATs include AT4_FkbB_ from FK506 and FK520 PKS, AT4_TiA2_ from fidaxomicin PKS, AT3_lsd12_ from lasalocid PKS, AT5_monAIV_ from monensin PKS and their mutants. However, the concentration of allmal- or ethmal-CoA was changed to 2 mM and other conditions were unchanged in the reactions of AT4_TiA2_ and its mutants in fidaxomicin PKS. holo-ACPs include holo-ACP2/4_FkbB_ and ACP10_FkbA_. Allylmalonylation and ethylmalonylation conversion yields in *trans*-acylation reactions, comparing ion peak heights of acylated and intact protein in MS, were selected the same to HPLC.

### HPLC–MS Analysis for Acyl-Transfer

The reaction mixtures were analyzed on HPLC–MS equipped with an Agilent 1200 HPLC system (Agilent, Santa Clara, CA, United States) and a Thermo Finnigan LCQDeca XP Max LC/MS system (Thermo Finnigan, Waltham, MA, United States). HPLC separation was performed on an Agilent SB-C18 column (3.5 um particle size, 80 Å, 2.1 mm × 150 nm) at 35°C. Solvent A was water containing 0.1% formic acid. Solvent B was acetonitrile. HPLC condition for *self*-acylation reaction: a linear gradient from 90 to 70% solvent A from 0 to 5 min, a linear gradient from 70 to 60% solvent A from 5 to 40 min, a linear gradient from 60 to 50% solvent A from 40 to 60 min, a linear gradient from 50 to 30% solvent A from 60 to 70 min. While, HPLC condition for *trans*-acylation reaction: a linear gradient from 90 to 70% solvent A from 0 to 5 min, a linear gradient from 70 to 50% solvent A from 5 to 55 min, a linear gradient from 50 to 30% solvent A from 55 to 60 min. The equilibration to initial conditions was for 13 min at a flow rate of 0.2 mL/min. UV detection was performed at both 220 and 280 nm. MS with an electrospray ionization (ESI) source was performed as follows: positive mode, source voltage of 2.5 kV, capillary voltage of 41 V, sheath gas flow of 45 arbitrary units, auxiliary/sweep gas flow of 5 arbitrary units, capillary temperature 330°C.

### Docking Models and Molecular Dynamics Simulations (MDs)

The homology models of [KS4][AT4]_FkbB_ and ACP4_FkbB_ of FK506 PKS were constructed by SWISS-MODEL^1^. Before molecular docking, the proteins were prepared using 3Drefine, which added hydrogen atoms and optimized atomic-level energy to consistent protein structure refinement. The structures of allmal and ethmal were constructed by Discovery Studio 2.5 (Accelrys Software, Inc., San Diego, CA, United States). The resulting [KS4][AT4]_FkbB_ model was used as a starting point for docking the allmal/ethmal substrate into AT4_FkbB_ using UCSF DOCK^2^. The best resulting model was selected on the basis of the docking scores and the conservation of key interactions that support the specificity of the enzyme (Dunn et al., 2013) and the predicted free binding energy. MDs of both allmal/ethmal-[KS4][AT4]_FkbB_ and allmal/ethmal-[KS4][Q119A-L185I-V186D-V187T-F203L]_FkbB_ complexes were performed by AMBER^3^.

### Preparation of P1-Derived Artificial Chromosome (PAC) Library

Mycelia of *S.* *tsukubaensis* YN06 was used to construct a PAC library using a modified *E. coli*–*Streptpmyces* Artificial Chromosome (ESAC) vector by Bio S&T (Montreal, QC, Canada). The pESAC13 vector contains a phiC31 integrase gene *oriT* and a phiC31 *attP* site that permit conjugation into *Streptomyces* strains and integration at the *attB* recombination locus. The average insert mycelia DNA size of the pESAC library was estimated to be 125 kb (Jones et al., 2013). PAC-B18 was identified by PCR screening using primers 61–66 to contain the entire FK506 gene cluster and it was chosen for heterologous expression.

### AT4_FkbB_ Site-Directed Mutagenesis of *S.* *tsukubaensis* YN06 PAC

The process that PAC-B18 containing AT4_FkbB_ in the FK506 gene cluster was mutated from amino acid 187 Val (GTC) to Lys (AAG), is depicted in **Supplementary Figure S1**. Briefly, the PAC-B18 plasmid was electroporated into *E. coli* DH10β harboring pSC101-*ccdA*-*gbaA*. CcdA is as antidote for the counterselectable agent CcdB, which expressed to be toxic to *E. coli* (Wang et al., 2014). The linear targeting molecule containing *ccdB*-*amp* was amplified from p15A-*ccdB*-*amp* by PCR using primers 67–68. The first recombinants were cultured on LB plates containing ampicillin and analyzed by sequence. In the second round of recombineering, the linear targeting AT4_FkbB_ fragment was amplified from pET28a-V187K using primers 69–70 for electroporation. The second recombinants were incubated on LB plates at 37°C overnight and analyzed by sequence using primers 69–70. The resulting recombinant was named as PAC-B18-V187K in following experiments.

### Introduction of PACs Into *S.* *tsukubaensis* YN06 and Fermentation

The *E. coli* DH10β containing PAC-B18 or PAC-B18-V187K (Apra^R^) was used in a dipparental mating with the other *E. coli* strain ET12567 that contained the RP4 derivative pUZ8002 (Kan^R^) respectively (**Supplementary Figure S2**). The method of dipparental mating was accoding to Jones et al. (2013) to obtain the ET12567 strains containing PAC-B18 or PAC-B18-V187K (Apra^R^) and pUZ8002 (Kan^R^). Then the obtained ET12567 strains were conjugated with the mycelia on ISP4 medium with 20 mM MgCl_2_ and 50 mM CaCl_2_ after 20 h with 20 μg/ml apramycin and 25 μg/ml nalidixic acid for 6–9 days at 28°C. Exconjuants were streaked on ISP4 plates with 20 μg/ml apramycin and further confirmed by PCR amplification, T-vector construction, and sequenced using the primers 69–70. The resulting *S. tsukubaensis* strains YN06-01 (PAC-B18), YN06-02 (PAC-B18-V187K) and wild type were grown on ISP4 plates for 5–9 days at 28°C to allow colonies to sporulate for fermentation.

For fermentation, an agar piece of approximately 1 cm^2^ was inoculated into a 250-ml flask containing 50 ml of the seed medium, consisting of 2% TSB and 5% PEG6000, and was maintained at 28°C and 220 rpm for 24 h. The seed culture to final concentration of OD_600_ = 0.4 was inoculated into a 250-ml flask containing 30 ml of the fermentation medium (5% maltodextrin, 1% yeast extract, 3% cotton seed meal, 0.2% K_2_HPO_4_, 0.1% CaCO_3_, pH 6.8). The culture condition was maintained at 28°C and 220 rpm for 5 days (Wang et al., 2016).

### Detection of FK506 and FK520

Culture samples (2 ml) from each *S. tsukubaensis* strain were withdrawn and ultrasonic extracted with 500 μl of methanol. Methanol layer was recovered by centrifugation at 12000 rpm for 15 min. The concentration of FK506 and FK520 was determined using an HPLC system (Agilent Series 1100, Agilent) equipped with a SB-C18 column (150 mm × 2.1 mm, Agilent). The column temperature was maintained at 60°C and UV detector was set at 215 nm. The mobile phase, which had a flow rate of 1.0 mL/min, contained 0.1% H_3_PO_4_ solution (A) and acetonitrile (B). HPLC condition: a linear gradient from 40 to 70% solvent B from 0 to 10 min, a linear gradient at 70% solvent B from 10 to 35 min. The FK506 and FK520 titers were calculated with standard curves.

## Results

### Preference of AT4_FkbB_ on Allmal-CoA Transfer

**FIGURE 1:**
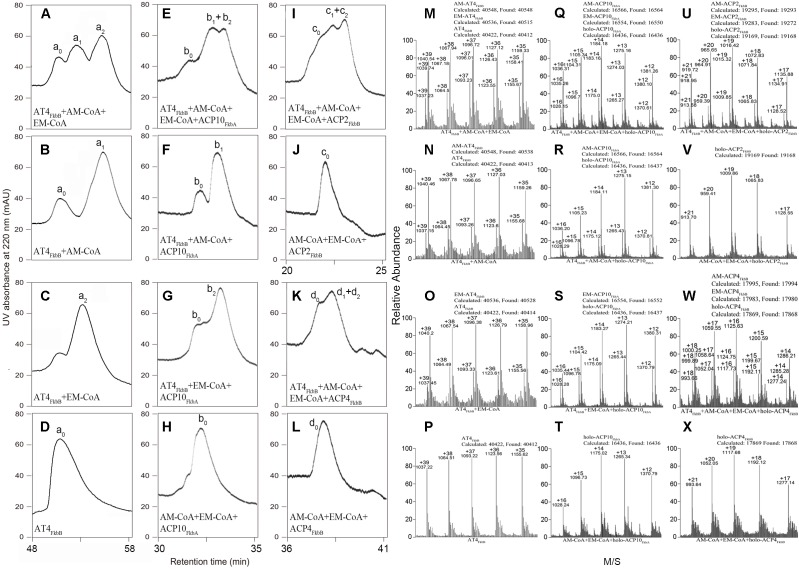
HPLC and MS analyses of AT4_FkbB_ recognizing and transferring allmal- and ethmal-CoA in *self*-acylation reactions and onto diverse ACPs in *trans*-acylation reactions. **(A–C,M–O)** HPLC and MS analyses of incubation of AT4_FkbB_ with allmal-CoA : ethmal-CoA = 1:1 (A, M), allmal-CoA **(B,N)**, ethmal-CoA **(C,O)**. **(D,P)** HPLC and MS analyses of AT4_FkbB_. **(E–G,Q–S)** HPLC and MS analyses of transferring allmal-CoA : ethmal-CoA = 1:1 **(E,Q)**, allmal-CoA **(F,R)**, ethmal-CoA **(G,S)** to holo-ACP10_FkbA_ in the presence of AT4_FkbB_. **(H,T)** HPLC and MS analyses of incubation of holo-ACP10_FkbA_ with allmal-CoA : ethmal-CoA = 1:1. **(I,J,U,V)** HPLC and MS analyses of transferring allmal-CoA : ethmal-CoA = 1:1 to holo-ACP2_FkbB_ in the presence of AT4_FkbB_ **(I,U)** and in the absence of AT4_FkbB_ **(J,V)**. **(K,L,W,X)** HPLC and MS analyses of transferring allmal-CoA : ethmal-CoA = 1:1 to holo-ACP4_FkbB_ in the presence of AT4_FkbB_ **(K,W)** and in the absence of AT4_FkbB_ **(L,X)**. The peaks were assigned as follows: a_0_, AT4_FkbB_; a_1_, allmal-AT4_FkbB_; a_2_, ethmal-AT4_FkbB_; b_0_, holo-ACP10_FkbA_; b_1_, Allmal-ACP10_FkbA_; b_2_, ethmal-ACP10_FkbA_; c_0_, holo-ACP2_FkbB_; c_1_, allmal-ACP2_FkbB_; c_2_, ethmal-ACP2_FkbB_; d_0_, holo-ACP4_FkbB_; d_1_, allmal-ACP4_FkbB_; d_2_, ethmal-ACP4_FkbB_.

We have reported that AT4_*FkbB*_ in FK506 PKS can transfer both allmal and ethmal units to ACP4_FkbB_, accounting for simultaneous production of FK506 and FK520 in *S.* *tsukubaensis* YN06 (Jiang et al., 2015). To further understand the possible competition between allmal and ethmal units to AT4_*FkbB*_ in *self*-acylation reactions, equal molar ratio of allmal- and ethmal-CoA was added to AT4_*FkbB*_ *in vitro* assays. HPLC data showed that a new large peak appeared along with AT4_FkbB_ (**Figures 1A–D**), and this HPLC peak contained two products, which were identical to the biochemically synthesized allmal- and ethmal-AT4_FkbB_ based on mass spectrum (MS) data, indicating *self*-acylation has occurred on AT4_FkbB_. Allylmalonylation and ethylmalonylation conversion yields were estimated to be 46.4 and 38.6%, respectively, by comparing the ion peak strength of both acylated and un-acylated proteins on MS which selected was the same to the retention time of HPLC (Jiang et al., 2015; Wang Y.Y. et al., 2015) (**Figures 1M–P**). These data suggested that allmal-CoA is the preferred substrate for AT4_FkbB_ in formation of intermediates in the *self*-acylation reactions.

To further discover the competition of allmal and ethmal units onto the acyl receptor ACPs in *trans*-acylation reactions, equal molar of allmal and ethmal units were incubated with AT4_*FkbB*_ and different holo-ACPs, including holo-ACP2_FkbB_, holo-ACP4_FkbB_, and holo-ACP10_FkbA_ (ACP in the 2nd, 4th, and 10th module of FK506 PKS to accept MM units, both allmal and ethmal units, M unit, respectively). We observed appearance of one or two new peaks with all holo-ACPs (**Figures 1E–G,I,K**), compared to control reactions without AT4_FkbB_ (**Figures 1H,J,L**). Both the retention time and the MS data confirmed that these peaks were allmal- and ethmal-ACPs (**Figures 1E–L,Q–X**). The acylation ratios of allmal and ethmal onto ACP2_FkbB_, ACP4_FkbB_, and ACP10_FkbA_ were 44.3 and 37.9% (**Figure 1Q**), 42.1 and 35.2% (**Figure 1U**), 44.4 and 37.6% (**Figure 1W**), respectively. These results suggested that allmal-CoA is preferred over ethmal-CoA as a substrate for AT4_FkbB_ in transferring the acyl group to diverse ACPs in the *trans*-acylation reactions, and that the specificity of acyl groups is determined by AT4_FkbB_ rather than ACPs. And ACP10_FkbA_ was chosen as an acyl receptor in the biochemical assays below.

### Gln119, Leu185-Val186-Val187, and Phe203 Residues Are Critical for Allmal Transfer by AT4_FkbB_

**FIGURE 2:**
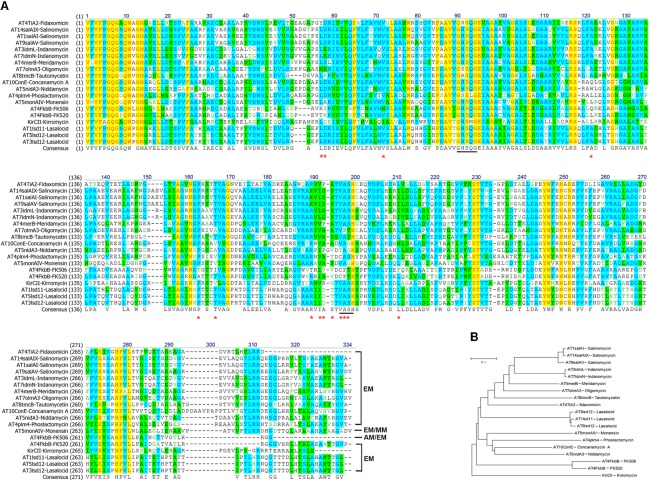
Amino acid sequence alignments and phylogenetic tree of AT4_FkbB_ of FK506 PKS and ethmal-specific ATs. **(A)** Amino acid sequence alignments between AT4_FkbB_ and ethmal-specific ATs. Sequences colored in yellow and blue or green were corresponded to the results of identify positions and consensus positions. The residues in AT4_FkbB_ labeled by red stars were mutated to the correspondingly conserved residues in ethmal-specific ATs. The residues labeled by black line marks were the catalytic centers of ATs. **(B)** Phylogenetic tree of AT4_FkbB_ and ethmal-specific ATs. A phylogenetic tree was generated using Mega 5.05 neighbor-joining method. ATs are shown as the locations in PKSs and names of corresponding polyketides in parenthesis. The selection of 18 ethmal-specific ATs were from PKSs of FK520, fidaxomicin, lasalocid, monensin, kirromycin, phoslactomycin, meridamycin, salinomycin, indanomycin, oligomycin, tautomycetin, concanamycin A, niddamycin, and phoslactomycin.

Our above results suggested that allmal-CoA was preferred over ethmal-CoA by AT4_*FkbB*_ in both *self*- and *trans*-acylation reactions for FK506 biosynthesis. Next, protein alignment of AT4_FkbB_ with 18 ethmal-specific ATs from other diverse PKSs was demonstrated that 14 residues (Lys54-Ser55, Met69, Gln119, Thr156, Ile160, Thr183, Leu185-Val186-Val187, Cys189-Pro190-Thr191, and Phe203) of AT4_FkbB_ might be involved in the discriminative recognition of allmal- and ethmal groups, since residues in these positions from ethmal-specific ATs are highly conserved (**Figure 2A**). Then all these residues in AT4_FkbB_ were mutated to the corresponding AAs in ethmal-specific ATs individually, and all the mutant proteins were expressed and purified from *E*. *coli* for *in vitro* biochemical assays.

Notably, both Q119A and L185I-V186D-V187T mutants showed less than half *self*-acylation activities of wild type AT4_FkbB_ with allmal-CoA, and dramatically decreased *trans*-acylation activities by 28 and 36%, respectively. However, these mutants had no significant difference in enzymatic activities with ethmal-CoA as the substrate in either *self*- or *trans*-acylation reactions (**Table 1**). Additionally, F203L mutant of AT4_FkbB_ led to dramatic decrease in *trans*-acylation activity with allmal-CoA as the substrate. However, it had a higher *trans*-acylation activity with ethmal-CoA as the substrate (**Table 1**). These results suggested that mutations of Q119A and L185I-V186D-V187T might have negative effects on allmal-AT4_FkbB_ intermediate formation during *self*-acylation reactions, leading to less allylmolonylation of ACP10_FkbA_ in *trans*-acylation reactions, while F203L has an important role on allmal transfer to ACP10_FkbA_ in *trans*-acylation reaction. The other 11 mutants displayed similar acylation activities to the wild type (**Table 1**), suggesting that the residues at these positions had no significant impacts on AT4_FkbB_ in recognizing and transferring the allmal unit to ACP10_FkbA_. A Q119A-L185I-V186D-V187T-F203L mutant was further constructed and displayed a lower (<16%) *trans*-acylation activity with allmal-CoA but a higher activity with ethmal-CoA (**Table 1**). Furthermore, all 15 mutants had very similar circular dichroism (CD) spectra to the wild type AT4_FkbB_, suggesting that they had similar secondary structures (**Supplementary Table S1**). Cumulatively, these data suggested that residues Gln119, Leu185-Val186-Val187, and Phe203 of AT4_FkbB_ are crucial for determining the substrate specificity.

**Table 1:** The activities of AT4_FkbB_ and its mutants of Fk506 PKS with allmal- or ethmal-CoA as the substrate in *self*- and *trans*-acylation reactions.

Mutant	*Self*-acylation reaction (%)		*Trans*-acylation reaction (%)	
	Allmal-AT4^∗^_FkbB_	Ethmal-AT4^∗^_FkbB_	Allmal-ACP10_FkbA_	Ethmal-ACP10_FkbA_
AT4_FkbB_	69.7	62.0	75.1	63.7
K54L-S54D	65.5	63.1	70.7	74.0
M69V	68.5	60.6	86.3	82.2
Q119A	**29.2**	65.4	**27.1**	61.6
T156P-I160T	69.8	70.2	54.5	81.8
T183R	65.0	62.9	75.3	67.7
L185I-V186D-V187T	**28.0**	65.8	**21.3**	71.7
F203L	64.3	65.1	**38.8**	73.1
C189V-P190A-T191S	66.5	64.6	79.0	75.4
Q119A-L185I-V186D-V187T-F203L	**27.1**	67.8	**12.1**	81.5
S89C	44.7	45.1	70.5	82.8
S89C-F203L	56.2	51.1	35.7	78.3
V187A	33.4	36.1	34.6	40.8
V187D	35.6	30.3	40.3	28.7
V187C	45.6	31.2	52.2	34.9
V187I	45.9	30.8	48.2	37.0
V187K	**78.8**	**32.3**	**93.4**	**46.4**
V187W	52.4	29.4	66.8	48.6
V187F	38.9	31.2	45.6	36.2

*The bold values represent the significant change in activities.*

### Structure Modeling-Based Analysis of Gln119, Leu185-Val186-Val187, and Phe203 for Substrate Specificity of AT4_FkbB_

**FIGURE 3:**
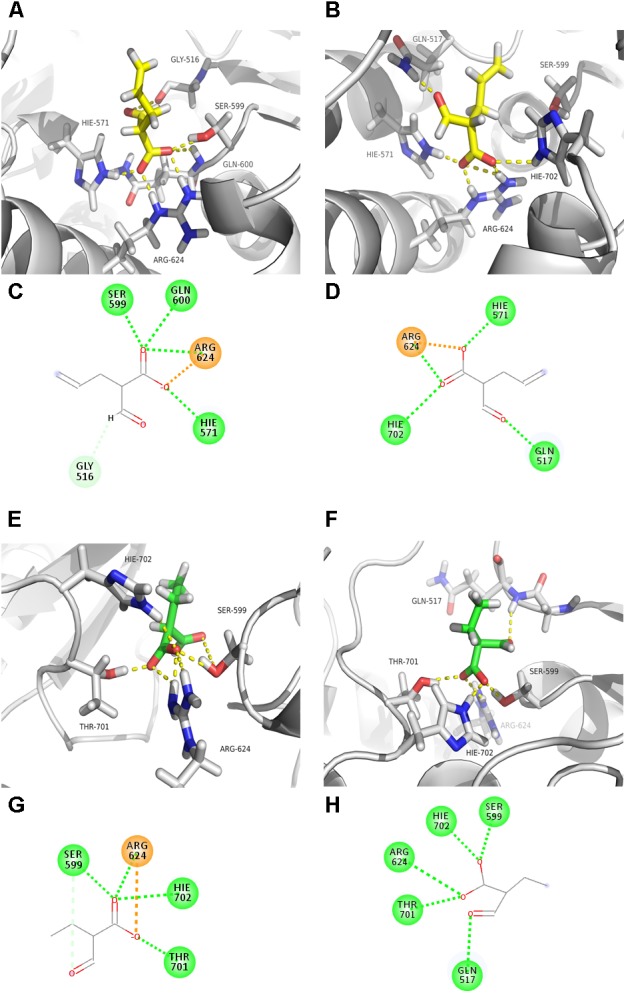
Molecular interactions between the binding pocket of AT4_FkbB_ and its mutant Q119A-L185I-V186D-V187T-F203L with allmal or ethmal unit. Structure models of AT4_FkbB_ **(A)** or mutant **(B)** with bound allmal unit. Molecular interactions between residues in AT4_FkbB_ **(C)** or mutant **(D)** with the oxygen atoms of allmal group. Structure models of AT4_FkbB_ **(E)** or mutant **(F)** with bound ethmal unit. Molecular interactions between residues in AT4_FkbB_ **(G)** or mutant **(H)** with the oxygen atoms of ethmal group. Residues, allmal unit, ethmal unit, hydrogen and oxygen atoms were colored gray, yellow, green, dark blue, and red. The figures were made by PyMOL. The dotted green, orange, and soft blue lines between residues and the oxygen atoms of allmal- or ethmal group represented the conventional hydrogen bonds, the attractive charges and the carbon hydrogen bonds.

To further uncover the potential mechanism of residues Gln119, Leu185-Val186-Val187, and Phe203 in substrate specificity of AT4_FkbB_, a homology model of [KS4][AT4]_FkbB_ with ethmal or allmal unit bound to its active site was constructed. The crystal structures of [KS3][AT3] (2QO3A) (Tang et al., 2007) and [KS5][AT5] (2HG4A) (Tang et al., 2006) of DEBS were used as the templates to establish a reliable [KS4][AT4]_FkbB_ structural model, because they all shared high sequence identity with [KS4][AT4]_FkbB_ (>40% of sequence identity, data not shown). The resulting structure model was used for docking of allmal or ethmal into the proposed active sites. Then the dockings of allmal- and ethmal-[KS4][AT4]_FkbB_ were modeled by MDs for assessment of the quality of the wild type models. As expected, a hydrogen bond was observed from MDs between the hydrogen atom in the Ser599 hydroxyl group within the conserved GHSXG motif of [KS4][AT4]_FkbB_ and the unprotonated nitrogen atom of the conserved His702 in the motif CPTH (H_599_...N_702_ distance is 3.0 ± 0.1 Å), indicating the roles of His702 in activating the catalytic residue Ser599 on nucleophilic attack on the allmal or ethmal thioester (Ser599 and Ser89 are the same site in [KS4][AT4]_FkbB_ and AT4_FkbB_, respectively). And the distance between the nucleophilic hydroxyl oxygen atom of Ser599 (O) and the electrophilic carbonyl carbon atoms along with side chain carboxyl of allmal (C) and ethmal (C) were 3.3 ± 0.2 and 3.2 ± 0.2 Å, respectively (**Figures 3A,C,E,G**), indicating the possible formation of the pre-reactive complexes of allmal- and ethmal-[KS4][AT4]_FkbB_.

Next, the dockings of mutant [KS4][Q119A-L185I-V186D-V187T-F203L]_FkbB_ with allmal or ethmal were modeled by MDs. The results showed that in the model of allmal-[KS4][Q119A-L185I-V186D-V187T-F203L]_FkbB_, the distance between the nucleophilic hydroxyl oxygen atom of Ser599 (O) and the carbonyl carbon atom along with side chain carboxyl of allmal (C) was 5.1 ± 0.2 Å (**Figures 3B,D**), although the interactive pattern between the binding pocket with allmal in the mutant protein was similar to the wild type [KS4][AT4]_FkbB_. It suggested that extended distance between Ser599 and AM possibly leads to less formation of allmal-enzyme intermediate. In contrast, in the model of ethmal-[KS4][Q119A-L185I-V186D-V187T-F203L]_FkbB_, the ethmal-thioester was kept in a position still susceptible attacked by Ser599 for the production of the ethmal-enzyme intermediate (**Figures 3F,H**). Our results suggested that mutations (Q119A, L185I, V186D, V187T, and F203L) reduced allmal specificity due to less nucleophilic attacks between Ser599 and allmal for formation of the allmal-enzyme intermediate.

### Enhanced Allmal Transfer by Mutations of Crucial Residues in Ethmal-Specific ATs

Our above results suggested that the residues Gln119, Leu185-Val186-Val187, and Phe203 in AT4_FkbB_ of FK506 PKS were critical for transfer of allmal unit. To further validate this hypothesis, a series of mutants were constructed in ethmal-specific ATs, including AT4_FkbB_ of FK520 PKS, AT4_TiA2_ in fidaxomicin PKS, AT3_lsd12_ of lasalocid PKS and AT5_monAIV_ of monensin PKS, which were chosen based on the phylogenetic tree (**Figure 2B**). The mutant versions of AT4_FkbB_ of FK520 PKS (L145Q, I211L-A212V, and L229F), AT4_TiA2_ of fidaxomicin PKS (A120Q, V186L-P187V-A188V, and L204F), AT3_lsd12_ of lasalocid PKS (A119Q, I186L-D187V-T188V, and L204F), and AT5_monAIV_ of monensin PKS (L119Q, V186L-A187V-S188V, and L204F) (**Figure 2A**) were expressed and purified from *E. coli* for biochemical assays. Three mutants L145Q, I211L-A212V, and L229F of AT4_FkbB_ in FK520 PKS showed higher activities in *self-* and *trans*-acylation reactions with allmal-CoA as a substrate by 61.3 and 31.6, 58.2 and 35.8, 50.1 and 13.1%, respectively, but no altered *self*- and *trans*-acylation activities with ethmal-CoA as a substrate compared with wild type AT4_FkbB_ of FK520 PKS (**Table 2**). The results suggested that mutations of L145Q, I211L-A212V, and L229F in AT4_FkbB_ of FK520 PKS enhanced acyl unit specificity on allmal unit. Similar results were observed in mutants of AT4_TiA2_, AT3_lsd12_, and AT5_monAIV_ (**Table 2**), further confirming the essentiality of these five residues for allmal specificity.

**Table 2:** The activities of AT4_FkbB_ of FK520 PKS, AT4_TiA2_ of fidaxomicin PKS, AT3_lsd12_ of lasalocid PKS and AT5_monAIV_ of monensin PKS and their mutants with allmal- or ethmal-CoA as the substrate in *self*-acylation reactions and with allmal- or ethmal-, M-, MM-CoA as the substrate in *trans*-acylation reactions.

Mutant	*Self*-acylation reaction (%)		*Trans*-acylation reaction (%)			
	Allmal-AT4_FkbB_^∗^	Ethmal-AT4_FkbB_^∗^	Allmal-ACP10_FkbA_	Ethmal-ACP10_FkbA_	M-ACP10_FkbA_	MM-ACP10_FkbA_
AT4_FkbB_	31.5	65.1	10.2	69.1	2.1	0.4
L145Q	61.3	63.2	31.6	64.3	4.3	0.9
I211L-A212V	58.2	59.4	35.8	60.5	2.5	0.3
L229F	50.1	64.3	13.1	68.9	5.1	0.8
AT4_TiA2_	17.5	75.1	0.6	48.2	1.5	0.9
A120Q	30.9	59.3	13.9	41.3	1.2	0.8
V186L-P187V-A188V	31.4	61.2	18.4	37.3	0.4	0.8
L204F	22.8	52.6	4.4	49.2	1.1	1.2
AT3_lsd12_	17.8	78.1	0.8	78.2	0.4	0.6
A119Q	35.2	70.4	12.1	72.5	1.0	0.9
I186L-D187V-T188V	41.4	62.7	21.3	70.2	0.5	0.6
L204F	20.9	68.5	4.9	76.1	0.8	0.7
AT5_monAIV_	26.3	63.7	6.7	48.9	10.3	51.2
L119Q	42.1	61.2	21.9	45.6	9.7	48.7
V186L-A187V-S188V	49.8	50.1	25.7	39.2	11.2	28.6
L204F	33.6	55.2	14.8	49.9	16.3	45.6

Moreover, other commonly used acyl units, such as M- and MM-CoA, were used for specificity tests. Both acyl units were incubated with all above mutants and their corresponding wild type versions of ATs (AT4_FkbB_, AT4_TiA2_, AT3_lsd12_, and AT5_monAIV_) in *trans*-acylation reactions. LC–MS data showed that none of all mutants and their wild type counterparts had detectable *trans*-acylation activities with M- or MM-CoA, except that AT5_monAIV_ had activities with MM-CoA as previously reported (Pospisil et al., 1994; Leadlay et al., 2001), while the activities of its mutants to MM-CoA also remained (**Table 2**). Furthermore, the secondary structures of all mutants were similar to those of their wild type proteins as determined by CD spectra (**Supplementary Table S1**). These data further suggested that these five residues are specific for controlling allmal uploading by ATs.

### V187K of AT4_FkbB_ Enhancing Allmal Specificity in FK506 PKS

We showed the roles of residues Gln119, Leu185-Val186-Val187, and Phe203 in controlling allmal specificity of ATs. Next we attempted to enhance the substrate specificity of FK506 PKS AT4_FkbB_ on the allmal group. We found that three residues Leu185-Val186-Val187 had the most important roles in controlling allmal recognition by AT4_FkbB_ based on the allymalonylation conversion data (**Tables 1**, **2**). From the position of Leu695-Val696-Val697 with allmal, Val697 constrained the size of the allmal carbon chain to prevent more spacious polyketide building blocks such as ethmal, MM and M from incorporating to generate the diversely undesirable analogs (**Supplementary Figure S2A**).

**FIGURE 4:**
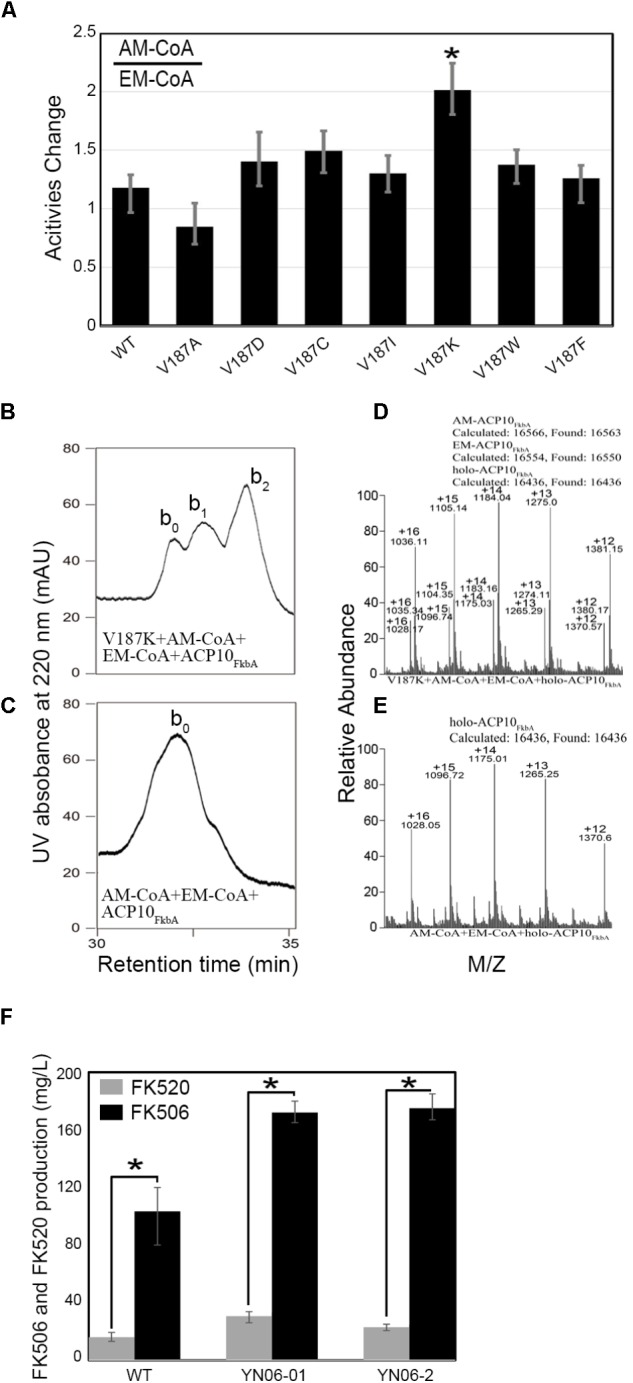
*Trans*-acylation reaction assays of various AT4_FkbB_ mutants after substitution of Val187 with Asp, or Cys, Ile, Lys, Trp, and Phe with allmal- and ethmal-CoA as substrates and the Production of FK506 and FK520 in WT, YN06-01, and YN06-02. **(A)** The change of transferring allmal/ethmal unit to ACP10_FkbA_ in *trans*-acylation reactions by AT4_FkbB_ and its mutants V187D, V187C, V187I, V187K, V187W, and V187F. HPLC analyses of transferring allmal-CoA : ethmal-CoA = 1:1 to holo-ACP10_FkbA_ in the presence of V187K **(B)** and in the absence of AT4_FkbB_ **(D)**. MS analyses of transferring allmal-CoA : ethmal-CoA = 1:1 to holo-ACP10_FkbA_ in the presence of V187K **(C)** and in the absence of AT4_FkbB_ **(E)**. The peaks were assigned as follows: b_0_, holo-ACP10_FkbA_; b_1_, allmal-ACP10_FkbA_; b_2_, ethmal-ACP10_FkbA_. **(F)** Production of FK506 and FK520 in WT, YN06-01 and YN06-02. Analyses of variance were conducted to determine the difference of WT and mutants using SPSS 20. The LSD multiple range tests were evaluated for significant differences among WT and mutants (*P* < 0.01). The data are expressed as mean ± SE (*n* = 3). ^∗^*P* < 0.01.

To test the important role of Val187 (Val697 and Val187 are the same site in [KS4][AT4]_FkbB_ and AT4_FkbB_ respectively) on allmal group recognition, V187A mutant was constructed and showed decreased *trans*-acylation activity to ∼60% of WT AT4_FkbB_ with allmal- or ethmal-CoA as the substrate as expected (**Table 1**). The mutants with substitution of Val187 with other 19 AAs in [KS4][AT4]_FkbB_ for docking of allmal or ethmal were modeled. The top scroes of AAs on enhancing the allmal specificity in 40 docking models were Asp, Cys, Ile, Lys, Trp, and Phe (**Supplementary Figure S2B**). Thereafter, mutants of Val187 to the six individual AAs in top scores were constructed for *in vitro* *self*- and *trans*-acylation reactions. We found that V187K mutant increased the ratio of allmal/ethmal from 1:1 to 2:1 (**Figure 4A**), while the overall activities of V187K were similar to WT in *trans*-acylation reactions (**Table 1**). The ratio of allmal/ethmal in other five mutants also increased (**Figure 4A**), but the overall activities were lower than WT (**Table 1**). To further test that V187K mutant had enhanced activities of allmal transfer, equal molar ratio of allmal and ethmal units were incubated with V187K mutant in *trans*-acylation reactions. HPLC data showed a new peak allmal-ACP10_FkbA_, larger than that of ethmal-ACP10_FkbA_ in size (**Figures 4B,C**), as confirmed by MS (**Figures 4D,E**). Allylmalonylation and ethylmalonylation conversion yields were estimated 57.2 and 28.3%, respectively, by comparing ion peak heights of acylated and intact proteins in MS (**Figures 4D,E**). These experiments suggested that targeted point mutation V187K in AT4_FkbB_ of FK506 PKS could enhance the substrate specificity for allmal unit.

Based on the biochemistry results of V187K, we introduced PAC-B18-V187K or PAC-B18 into *S. tsukubaensis* YN06 for the production of FK506 and FK520 (**Supplementary Figure S2**). The FK506 and FK520 production (172:29.1 mg/L) of strain YN06-01 (PAC-B18) in shake-flask fermentation had obvious change compared to that of *S. tsukubaensis* YN06 (103.3:17.0 mg/L). But the ratio of them in YN06-01 was the same to wild type about ∼6:1 (**Figure 4F** and **Supplementary Figure S3**), suggesting that the exogenous FK506 gene cluster PAC works in wild type. In strain YN06-02 (PAC-B18-V187K), the yield of FK506 and FK520 was 175.0 and 22.7 mg/L and the ratio of them was about ∼7.7:1 (**Figure 4F** and **Supplementary Figure S3**), suggesting V187K in AT4_FkbB_ of FK506 PKS could decrease the production of FK520.

## Discussion

Selection of the suitable acyl units in *self*- and *trans*-acylation reactions to load the starter or extending units for PKSs to produce diverse and biologically active natural products is dependent on ATs (Khosla et al., 2007; Dunn et al., 2013; Bravo-Rodriguez et al., 2014). Full understanding of interaction between ATs and the starter or extending units is required to overcome limitations related to the substrate specificity of ATs. Although several structural studies of ATs that utilize malonyl-CoA have been demonstrated (Liew et al., 2012), little is known regarding the specificity of ATs on unusual acyl-CoA. In this study, AT4_FkbB_ was selected as a model to analyze the recognition specificity on the extending units allmal and ethmal, since AT4_FkbB_ can transfer both allmal- and ethmal-CoA onto diverse ACPs in *self*- and *trans*-acylation reactions and allmal-CoA is preferred over ethmal-CoA as a substrate (**Figures 1E–L,Q–X**).

There are very few reports about the key AAs in ATs responsible for the intrinsic substrate specificity. Most studies focused on some sequence motifs from M- and MM-specific ATs (Haydock et al., 1995; Reeves et al., 2001; Vecchio et al., 2003). It was reported that replacement of Gln with Leu in the active site GHSQGE motif produced a mixture of 6dEB and the corresponding 6-desmethyl-6-dEB (Reeves et al., 2001). However, our results showed that allmal-specific motif GHSQGE in AT4_FkbB_ is the same to the most of ethmal-specific ATs. The M unit was incorporated after mutation of YASH to YAFH or HASH in AT6 of DEBS, where YASH and HAFH are the conserved sequence motifs for M and MM specificity, respectively (Vecchio et al., 2003). The motif CPTH in AT4_FkbB_ were mutated to ethmal-specific motif VASH, resulting in no altered enzymatic activities with allmal- and ethmal-CoA as substrates compared to WT (**Table 1**). Therefore, uploading of allmal or ethmal group into polyketide backbone is not dependent on the conserved motifs GHSQGE or CPTH in AT4_FkbB._ Gln119, Leu185-Val186-Val187, and Phe203 residues are newly identified residues in controlling allmal specificity, and confirmed by reverse mutations and functional analysis (**Tables 1**, **2**). Furthermore, Mutant Q119A-L185I-V186D-V187T-F203L transferred less allmal because of possible less nucleophilic attacks between the active site Ser599 and allmal unit as observed from MDs (**Figures 3B,D**). Here, we for the first time found that Gln119, Leu185-Val186-Val187, and Phe203 residues in AT4_FkbB_ were critical in determining the specificity toward allmal unit.

On Tab. 2, F203L mutant had a dramatically decreased *trans*-acylation activity with allmal unit, whereas the *self*-acylation activity was similar to WT, implying a potential role of F203L on the interaction between AT4_FkbB_ and ACP. But Phe203 did not interfere with the interaction between Ser89 of AT4_FkbB_ and phosphopantetheine attachment site Ser33 of ACP4_FkbB_ according to the homology model of ACP4_FkbB_ bound to the homology model [KS4][AT4]_FkbB_ and the cross-linking results (**Supplementary Figure S4**). How does F203L alter the substrate specificity? We speculated that F203L helped to allow ethmal group as a substrate to the active site Ser599, which was supported by the structural study of the M-AT_DY N_ covalent complex (Liew et al., 2012). F203L recognized sufficient allmal unit so that the *self*-acylation activity with allmal was similar to that of WT, if adequate reaction time was provided in the *self*-acylation reaction. When holo-ACP10_FkbA_, allmal-CoA, and F203L mutant were incubated together for 12 and 24 h, respectively, the activities of *trans*-acylation turned out similar to that of WT (data not shown), which partly confirmed our speculation. Therefore, further analysis of co-crystallizing F203L with allmal- or ethmal-CoA is still needed to discover the role of F203L in substrate specificity of AT4_FkbB_.

Gln119, Leu185-Val186-Val187, and Phe203 residues in AT4_FkbB_ determine allmal specificity as illustrated from MDs. Reverse mutations of these five residues in ethmal-specific ATs to the corresponding AAs of AT4_FkbB_ resulted in enhanced transfer of allmal onto ACP (**Table 2**). These data indicated that Gln119, Leu185-Val186-Val187, and Phe203 residues alter the substrate specificity of ethmal-specific ATs. The experimental data open up the possibility to control the substrate specificity in ATs by substitution of the critical residues, which will lead to the incorporation of non-native extending units for the production of ‘non-native’ natural products. Despite the protein engineering in ethmal-specific ATs for allmal specific transfer, the role of other residues on allmal or ethmal-specific recognition of ATs is not yet thoroughly elucidated. For this reason, determination of the three-dimensional structures of AT4_FkbB_ with allmal and ethmal units would substantially improve our understanding of the mechanism of other residues in allmal specificity of AT for efficiently governing FK506 biosynthesis.

## Author Contributions

Y-QL, X-MM, J-JS, and X-AC designed the experiments, analyzed the data, and wrote the manuscript. J-JS and X-FL performed the experiments. FC and X-XW performed dockings and MDs.

## Conflict of Interest Statement

The authors declare that the research was conducted in the absence of any commercial or financial relationships that could be construed as a potential conflict of interest.

## Footnotes

1 https://swissmodel.expasy.org/

2 http://dock.compbio.ucsf.edu/

3 http://ambermd.org/

## Abbreviations

AA: amino acid
ACPs: acyl carrier protein domains
allmal: allylmalonyl
AT: acyltransferase domain
ethmal: emthylmalonyl
FK506: tacrolimus
MDs: molecular dynamics simulations
PKS: polyketide synthase.
