# Supplementary figures and images for: Substrate Specificity of Acyltransferase Domains for Efficient Transfer of Acyl Groups
Source: Front Microbiol. 2018 Aug 7;9:1840. doi: 10.3389/fmicb.2018.01840 (PMC6090053; doi:10.3389/fmicb.2018.01840)

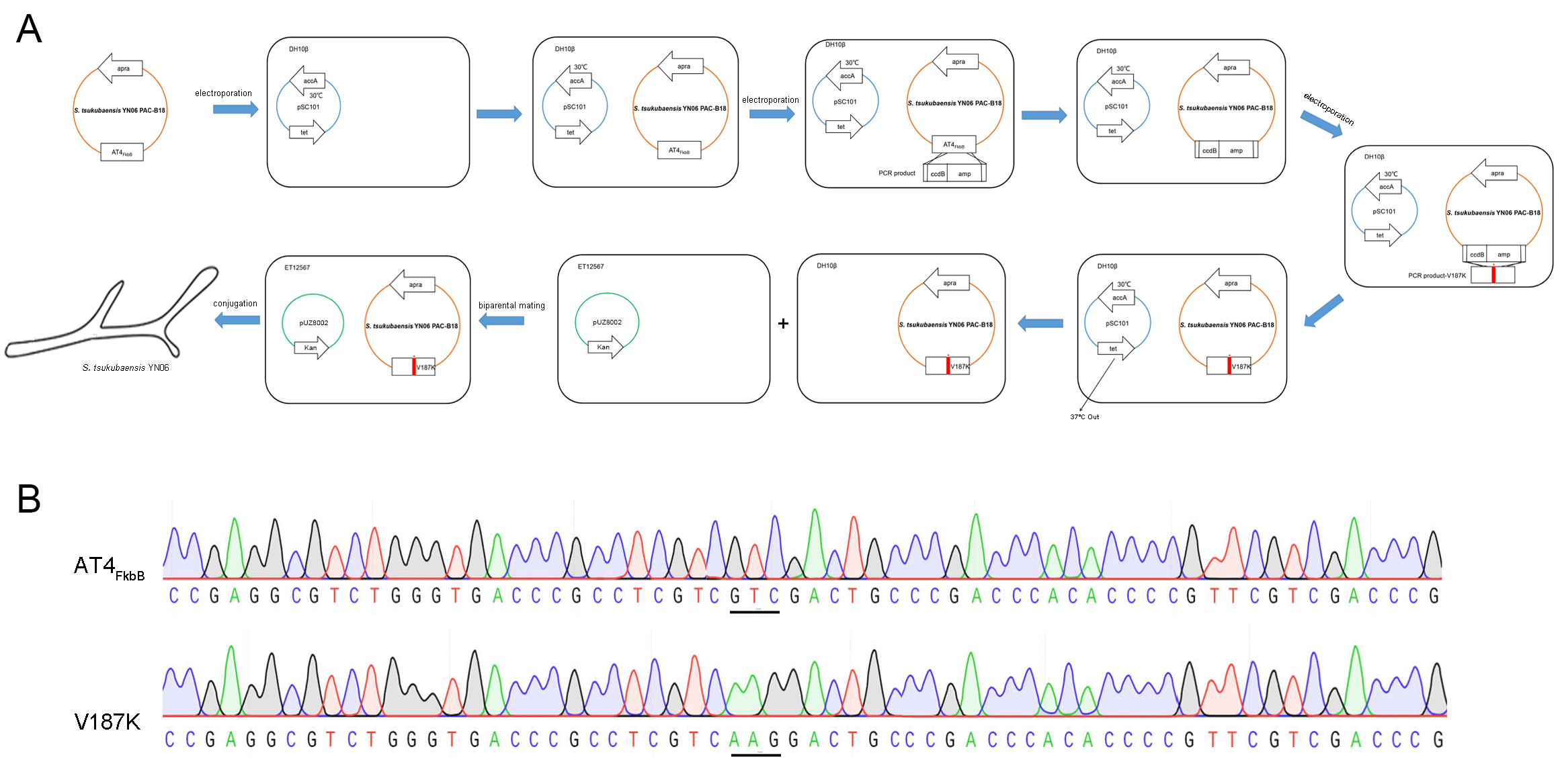

Supplement: Supplementary file 1 [file Image_1.TIF]

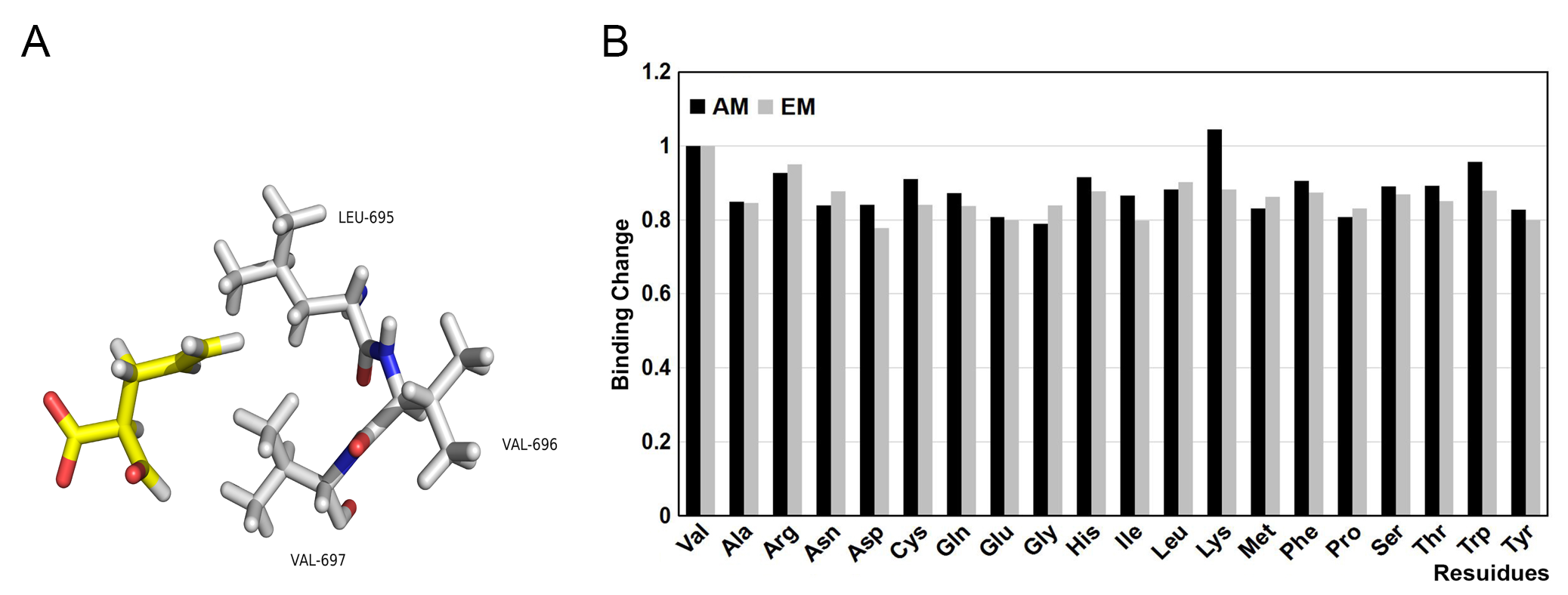

Supplement: Supplementary file 2 [file Image_2.TIF]

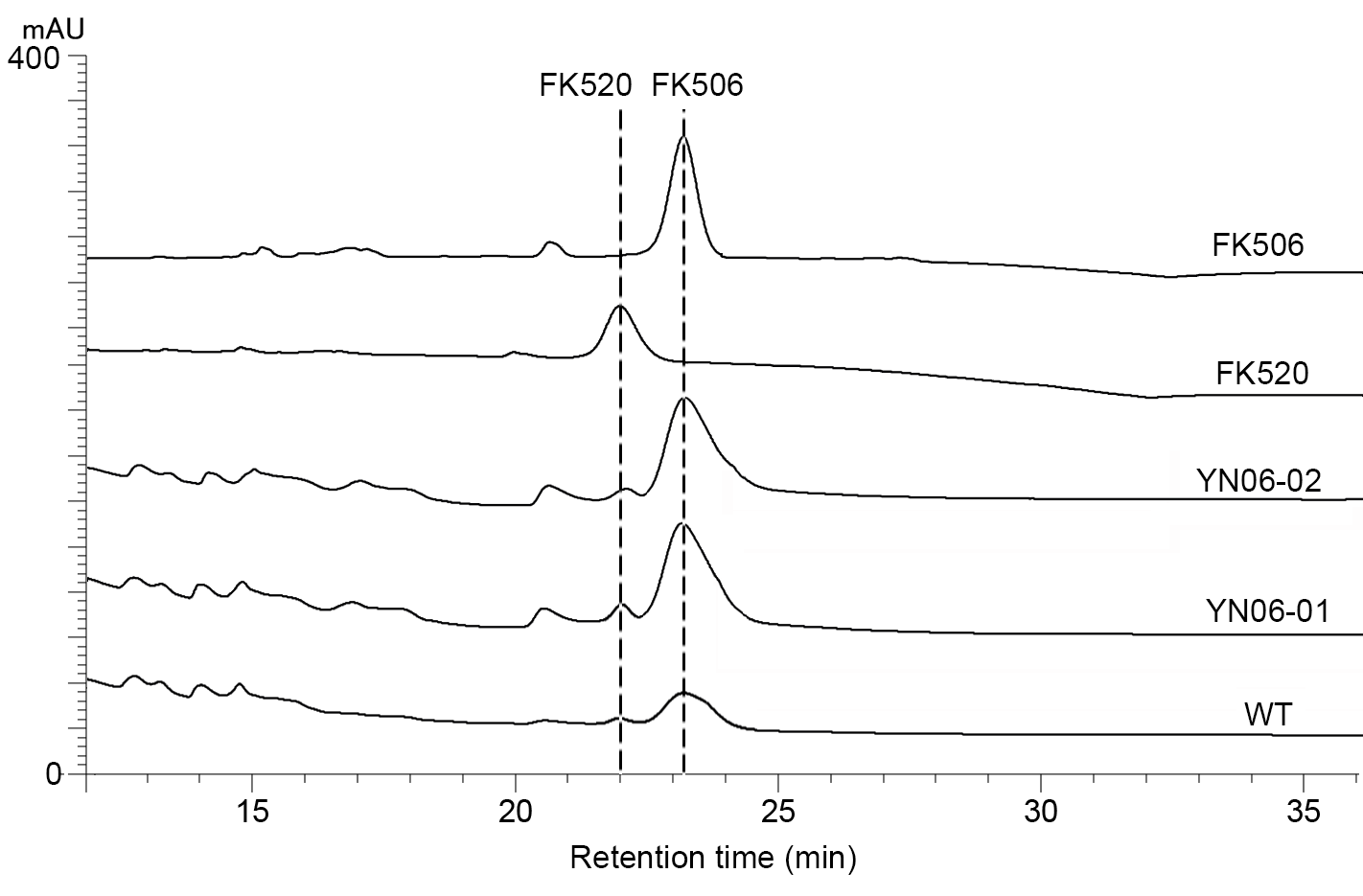

Supplement: Supplementary file 3 [file Image_3.TIF]

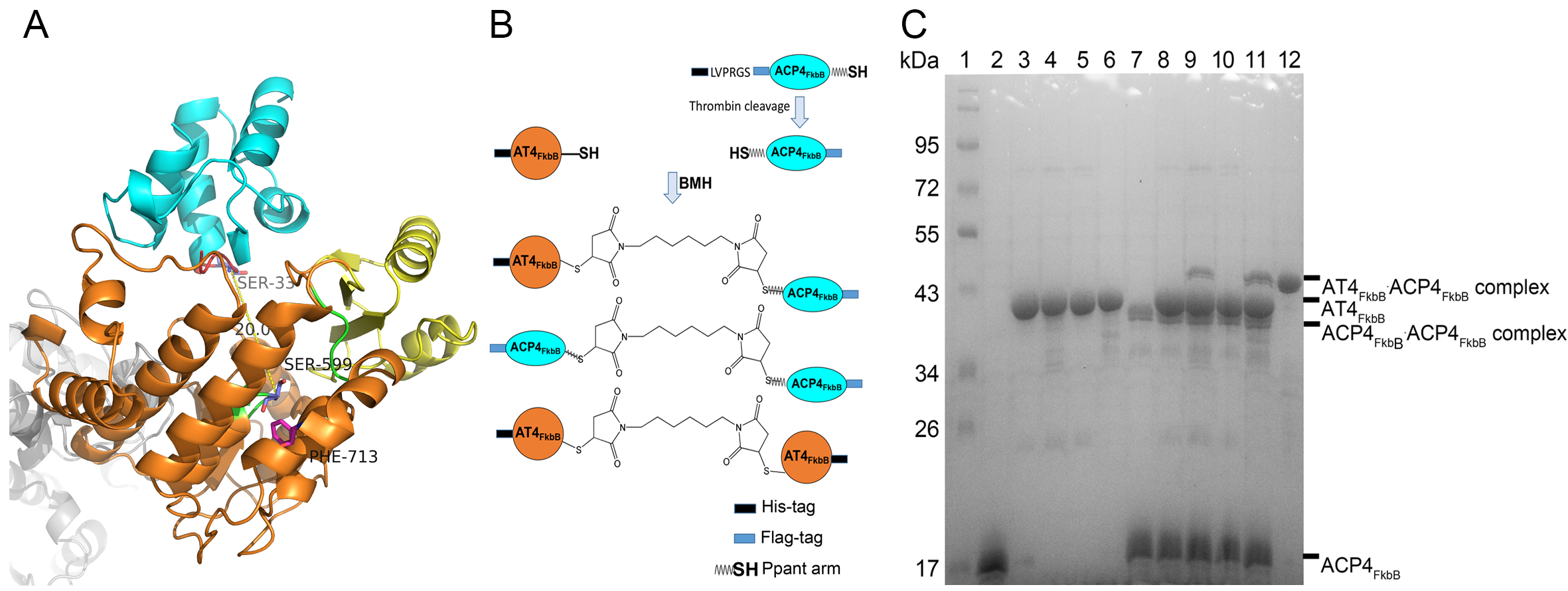

Supplement: Supplementary file 4 [file Image_4.TIF]
