# Supplementary material for: Substrate Specificity of Acyltransferase Domains for Efficient Transfer of Acyl Groups
Source: Front Microbiol. 2018 Aug 7;9:1840. doi: 10.3389/fmicb.2018.01840 (PMC6090053; doi:10.3389/fmicb.2018.01840)
Supplement: Supplementary file 5 [file Table_1.DOC]

**SUPPLEMENTARY MATERIALS**

**Table S1. The CD spectra of AT4_FkbB_ of FK506 and FK520 PKS, AT4_TiA2_ of fidaxomicin PKS, AT3_lsd12_ of lasalocid PKS and AT5_monAIV_ of monensin PKS and their mutants**

| **Mutant** | **Helix** | **Beta** | **Turn** | **Random** | **RMS Value** |
| --- | --- | --- | --- | --- | --- |
| AT4_FkbB_ of FK506 PKS | 21.7 | 44.6 | 2.6 | 31.1 | 13.704 |
| K54L-S55D | 32.8 | 29.9 | 5.7 | 31.6 | 13.293 |
| M69V | 27 | 39.5 | 3.9 | 29.6 | 12.994 |
| Q119A | 20.3 | 57.6 | 0.7 | 21.4 | 18.84 |
| T156P-I160T | 16.1 | 57.6 | 0 | 26.3 | 19.939 |
| T183R | 24.6 | 44.5 | 2.5 | 28.4 | 12.318 |
| F203L | 27.4 | 43 | 1.6 | 28 | 13.447 |
| L185I-V186D-V187T | 27.4 | 39.8 | 3.1 | 29.6 | 12.883 |
| C189V-P190A-T191S | 24.6 | 46.8 | 0.2 | 28.3 | 13.94 |
| Q119A-L185I-V186D-V187T-F203L | 24.6 | 44.4 | 2.5 | 28.5 | 12.319 |
| S89C | 18.4 | 56 | 1.6 | 24 | 19.006 |
| S89C-F203L | 18.3 | 55.6 | 1.8 | 24.3 | 18.839 |
| V187A | 18.1 | 55.1 | 2 | 24.7 | 18.674 |
| V187D | 18.1 | 55 | 2 | 24.8 | 18.679 |
| V187C | 26 | 43.8 | 2.0 | 28.2 | 12.882 |
| V187I | 21.5 | 44.8 | 2.6 | 31.1 | 13.705 |
| V187K | 27 | 39.4 | 4.0 | 29.6 | 12.995 |
| V187W | 16.1 | 57.6 | 0.3 | 26.0 | 19.937 |
| V187F | 24.7 | 44.3 | 2.6 | 28.4 | 12.321 |
| AT4_FkbB_ of FK520 PKS | 32.8 | 29.9 | 5.0 | 32.3 | 13.294 |
| L145Q | 27.0 | 39.5 | 3.9 | 29.6 | 12.994 |
| I211L-A212V | 30.3 | 47.6 | 0.7 | 21.4 | 13.015 |
| L229F | 29.9 | 34.7 | 4.4 | 31.0 | 13.144 |
| AT4_Tia2_ of fidaxomicin PKS | 25.3 | 49.8 | 0.0 | 24.9 | 11.280 |
| A120Q | 40.4 | 27.4 | 4.0 | 28.2 | 6.807 |
| V186L-P187V-A188V | 25.6 | 46.4 | 0.2 | 27.8 | 9.932 |
| L204F | 28.3 | 42.6 | 1.2 | 27.9 | 8.702 |
| AT3_lsd12_ of lasalocid PKS | 30.2 | 21.4 | 9.1 | 39.3 | 16.966 |
| A119Q | 13.9 | 53.7 | 2.8 | 29.5 | 19.15 |
| I186L-D187V-T188V | 30.1 | 21.5 | 9.1 | 39.3 | 16.967 |
| L204F | 13.7 | 54.9 | 3.1 | 28.3 | 20.472 |
| AT5monAIV of monensin PKS | 23.2 | 51.8 | 3.0 | 21.9 | 13.676 |
| L119Q | 18.7 | 59.9 | 0.0 | 21.4 | 19.984 |
| V186L-A1871V-S188V | 15.4 | 45.5 | 2.6 | 36.5 | 17.174 |
| L204F | 20.9 | 55.9 | 1.5 | 21.7 | 16.832 |

**Table S2. The primers used in this study**

| **Primer No.** | **Gene** | **Primer Sequence (**5'-3') |
| --- | --- | --- |
| P1 | Flag-tagged ACP4FkbB | GCCGCGCGGCAGCCATATGGATTACAAGGATGACGACGATAAGCCGGAGCGACTGCCGGACCT |
| P2 |  | CGAGTGCGGCCGCAAGCTTTCATCCCTTCCGTACATGCG |
| P3 | FK506-AT4_FkbB_-K54L-S55D | CCGGCCGTGCTCGACCGCGTCGACGTCGTCCAC |
| P4 |  | GTCGACGCGGTCGAGCACGGCCGGGTCTTCGAG |
| P5 | FK506-AT4_FkbB_-M69V | GCGGTCATGGTGTCCCTGGCCGCCGTGTGG |
| P6 |  | GGCCAGGGACACCATGACCGCCCAGCAGAC |
| P7 | FK506-AT4_FkbB_-Q119A | GCCCTGCTCGCCCGCGAACTCGCCGGACAC |
| P8 |  | GAGTTCGCGGGCGAGCAGGGCGGAGCGGAG |
| P9 | FK506-AT4_FkbB_-T156P-I160T | AACGGCCCCGGCACCACGACCGTGTCCGGGCGGCCCGAC |
| P10 |  | GGACACGGTCGTGGTGCCGGGGCCGTTGCGGCCCGCGAC |
| P11 | FK506-AT4_FkbB_-T183R | GTCTGGGTGCGACGCCTCGTCGTCGACTGC |
| P12 |  | GACGAGGCGTCGCACCCAGACGCCTCGGGC |
| P13 | FK506-AT4_FkbB_-L185I-V186D-V187T | GTGACCCGCATCGACACCGACTGCCCGACCCACACC |
| P14 |  | CGGGCAGTCGGTGTCGATGCGGGTCACCCAGACGCC |
| P15 | FK506-AT4_FkbB_-F203L | TACGACGAGCTGCAGCGGATCGCCGCGGCC |
| P16 |  | GATCCGCTGCAGCTCGTCGTACAGCGGGTC |
| P17 | FK506-AT4_FkbB_-C189V-P190A-T191S | GTCGTCGACGTGGCCAGCCACACCCCGTTCGTCGAC |
| P18 |  | CGGGGTGTGGCTGGCCACGTCGACGACGAGGCGGGT |
| P19 | FK506-AT4_FkbB_-S89C | ATCGGCCACTGCCAGGGGGAGATCGCCGCG |
| P20 |  | CTCCCCCTGGCAGTGGCCGATGACGGCGTC |
| P21 | FK506-AT4_FkbB_-V187A | CGCCTCGTCGCCGACTGCCCGACCCACACC |
| P22 |  | CGGGCAGTCGGCGACGAGGCGGGTCACCCA |
| P23 | FK506-AT4_FkbB_-V187D | CGCCTCGTCGACGACTGCCCGACCCACACC |
| P24 |  | CGGGCAGTCGTCGACGAGGCGGGTCACCCA |
| P25 | FK506-AT4_FkbB_-V187C | CGCCTCGTCTGCGACTGCCCGACCCACACC |
| P26 |  | CGGGCAGTCGCAGACGAGGCGGGTCACCCA |
| P27 | FK506-AT4_FkbB_-V187I | CGCCTCGTCATCGACTGCCCGACCCACACC |
| P28 |  | CGGGCAGTCGATGACGAGGCGGGTCACCCA |
| P29 | FK506-AT4_FkbB_-V187K | CGCCTCGTCAAGGACTGCCCGACCCACACC |
| P30 |  | CGGGCAGTCCTTGACGAGGCGGGTCACCCA |
| P31 | FK506-AT4_FkbB_-V187W | CGCCTCGTCTGGGACTGCCCGACCCACACC |
| P32 |  | CGGGCAGTCCCAGACGAGGCGGGTCACCCA |
| P33 | FK506-AT4_FkbB_-V187F | CGCCTCGTCTTCGACTGCCCGACCCACACC |
| P34 |  | CGGGCAGTCGAAGACGAGGCGTCGCACCCA |
| P35 | FK520-AT4_FkbB_-L145Q | GCGCTGCTGCAGCGTGAACTGGCGGGTCGT |
| P36 |  | CAGTTCACGCTGCAGCAGCGCGCTACGCAG |
| P37 | FK520-AT4_FkbB_-I211L-A212V | GTTCGTCGTCTCGTCGTTGATTGCCCGACCCAC |
| P38 |  | GCAATCAACGACGAGACGACGAACCCACACGCC |
| P39 | FK520-AT4_FkbB_-L229F | TATGACGAGTTCCAGCGTATCGTTGCGGAC |
| P40 |  | GATACGCTGGAACTCGTCATACAGCGGATC |
| P41 | Fidaxomicin-AT4_TiA2_ | GCCGCGCGGCAGCCATATGGGACCGCTGCCGTGGGTGCTC |
| P42 |  | CGAGTGCGGCCGCAAGCTTTTACTCCAGCCAGAACCGCTC |
| P43 | Fidaxomicin-AT4_TiA2_-A120Q | AAGCTGTTCCAGGAGGCGCTGGTCGGCAAC |
| P44 |  | CAGCGCCTCCTGGAACAGCTTGCTGCGCTC |
| P45 | Fidaxomicin-AT4_TiA2_-V186L-P187V-A188V | GCGCGGGTGCTCGTCGTCACCGTCGCGTCGCATTGC |
| P46 |  | CGCGACGGTGACGACGAGCACCCGCGCCTGCCAGCC |
| P47 | Fidaxomicin-AT4_TiA2_-L204F | CACGACCGGTTCGTCGGACTGCTCGACGGG |
| P48 |  | CAGTCCGACGAACCGGTCGTGCAGGGGATC |
| P49 | Lasalocid -AT3_lsd12_-A119Q | CAGCTGTTTCAGGATCATCTGACCGGTCAT |
| P50 |  | CAGATGATCCTGAAACAGCTGGCTACGCAG |
| P51 | Lasalocid -AT3_lsd12_-I186L-D187V-T188V | GCGCGTATCCTCGTCGTCACCGTGGCGAGCCACAGC |
| P52 |  | CGCCACGGTGACGACGAGGATACGCGCACGCGCACC |
| P53 | Lasalocid -AT3_lsd12_-L204F | CACGACCAGTTCCTGCACCTGCTGGCGGAT |
| P54 |  | CAGGTGCAGGAACTGGTCGTGCAGCGGTTC |
| P55 | Monensin-AT5_monAIV_-L119Q | GTTCTGCTGCAGCGTCAGCTGAGCGGTCGT |
| P56 |  | CAGCTGACGCTGCAGCAGAACGCTACGCAC |
| P57 | Monensin-AT5_monAIV_-V186L-A1871V-S188V | GCGCGTGCGCTCGTCGTCGATGTGGCGGGTCATGGT |
| P58 |  | CGCCACATCGACGACGAGCGCACGCGCACGCAGGCC |
| P59 | Monensin-AT5_monAIV_-L204F | CTGGATCAATTCACCGAGGGTCTGGCGGGT |
| P60 |  | ACCCTCGGTGAATTGATCCAGGATCGCGTC |
| P61 | PAC-FK506-Up-F | CGCTACCTCGCCCATACG |
| P62 | PAC-FK506-Up-R | ACGAACGACCCGAGCATAG |
| P63 | PAC-FK506-Md-F | CTCGAACCCGAAGTAGTAGGAG |
| P64 | PAC-FK506-Md-R | GCGGATACCCCGGAAGAC |
| P65 | PAC-FK506-Dd-F | GGAGGAACGAAACCGACTTG |
| P66 | PAC-FK506-Dd-R | AGAGGCTTGCCGCTGTCA |
| P67 | FK506-AT4FkbB-ccdB-amp-F | | CGCTACGAGGCCCGAGGCGTCTGGGTGACCCGCCTCGTCGGTGTGGTAGCTCGCGTATT | | --- | |
| P68 | FK506-AT4FkbB-ccdB-amp-R | ACAGCGGGTCGACGAACGGGGTGTGGGTCGGGCAGTCCTTTGATCTGAATTCGGATCCT |
| P69 | FK506-V187K Frag-F | GCGCGAACTCGCCGGACACG |
| P70 | FK506-V187K Frag-R | TCACCCGGCTCGCGGGCGG |

**Figure S1. Introduction of PAC-B18-V187K into *Streptomyces* *tsukubaensis* YN06.** (**A**) Overview of PAC-B18-V187K into *Streptomyces* *tsukubaensis* YN06. First, the target PAC is introduced into the host carrying the pSC101-ccdA-gbaA. Electro-competent cells are prepared and electroporated with a linear targeting molecule containing a ccdB-selectable marker (amp^R^) fusion gene. Correct recombinants are obtained by selection for amp resistance together with L-arabinose induction of CcdA expression and incubation at 30^o^C (to retain pSC101-ccdA-gbaA). In the next step, cells harboring pSC101-ccdA-gbaA and correctly integrated ccdB-amp are grown in LB medium plus amp and L-arabinose at 30^o^C. Shortly before electroporation with another linear targeting molecule for the counterselection step, L-rhamnose is added to the cultures to induce Red expression. After electroporation, the cells are incubated at 37^o^C without L-arabinose to eliminate the pSC101 plasmid and CcdA expression. The surviving cells must have eliminated CcdB expression. Then, the DH10β strain containing the FK506 PAC (Apra^R^) and ET12567 strain containing pUZ8002 helper plasmid (Kan^R^), are dipparentally mated on LB agar without antibiotics to obtain the ET12567 strain containing the FK506 PAC and the plasmid pUZ8002 (Apra^R^, Kan^R^). Then the mycelia and the obtained ET12567 strains were conjugated into ISP4 medium plates. (**B**) The sequence of PAC-B18-V187K and PAC-B18 DNA fragment. The bases labeled by black line marks were the mutation site.

**Figure S2. Position of Val697 in AT4_FkbB_ with allmal unit and binding change of substitution of Val187 with each other nineteen amino acids in allmal- or ethmal-AT4_FkbB_ docking models.** **(A)** Position of Leu695-Val696-Val697 in AT4_FkbB_ with allmal unit. Val697 might act in constraining the choice of the substituent at position of the enzyme-bound allmal unit thioester. Residues Leu695-Val696-Val697, allmal unit, hydrogen and oxygen atoms were colored gray, yellow, drak blue and red. The figure was made by PyMOL. **(B)** The binding change of Val187 mutated to each other nineteen amino acids in allmal- or ethmal-AT4_FkbB_ docking models. The signals from docking of wild type AT4_FkbB_ (Val) with allmal and ethmal unit were normalizes as 1. The black and gray columns represented the docking of AT4_FkbB_ and its mutants with allmal and ethmal group respectively.

**Figure S3. HPLC** **analyses of FK506 and FK520 from day 6 fermentation cultures of WT, YN06-01 and YN06-02.**

**Figure S4. Docking models and cross-linking of AT4_FkbB_ with ACP4_FkbB_. (A)** A docking model of [KS4][AT4]_FkbB_-ACP4_FkbB_. A homology model of ACP4_FkbB_ bound to the homology model [KS4][AT4]_FkbB_ was constructed. The temples used for ACP4_FkbB_ were 5HV8A (Vance et al., 2016) and 2JU1A (Alekseyev et al., 2007) because they shared 49% and 55% of sequence identity with ACP4_FkbB_ (data not shown). The resulting docking structure model [KS4][AT4]_FkbB_-ACP4_FkbB_ showed that the ACP4_FkbB_ protein had contact with the small and large subdomains of AT4_FkbB_ and located at the entrance of the substrate tunnel to the active site Ser599 of the larger subdomain. The distance between the phosphopantetheine attachment site Ser33 of ACP4_FkbB_ and [KS4][AT4]_FkbB_ Ser599 was 20±2 Å, consistent with the length of a fully extended phosphopantetheine arm, suggesting that the ACP-tethered phosphopantetheine extending into the AT active site makes the *trans*-acylation reaction possible, indicating a reliable homology structural model.ACP4_FkbB_ homology model (blue labels) was used as a ligand protein to dock with AT4_FkbB_ structure (the small subdomain colored yellow labels and the big subdomain colored orange labels). The part of homology model of KS4_FkbB_ and the linker between KS4_FkbB_ and AT4_FkbB_ were colored in gray. The active site Ser599 of AT and the pantetheinate attachment site Ser33 of ACP were colored in dark blue. The conserved motifs GHSQG and CPTH of AT4_FkbB_ were colored in green. The resuidue Phe203 was colored in fuchsia. H and O atoms were colored in dark blue and red, respectively. The figure was made by PyMOL. **(B)** Cross-linking reactions of AT and ACP with orthogonal tags. The AT-ACP complex can be separated from the two homodimers by a 2-step purification procedure. **(C)** Cross-linking of AT4_FkbB_ and its mutants with holo-ACP4_FkbB_. For cross-linking assays, a mixture contains 10 μM AT4_FkbB_ (or F203L, S87C, S87C-F203L) and 40 μM holo-ACP4_FkbB_ of FK506 PKS, 100 mM sodium phosphate pH 7.2 and 200 μM bis(maleimido)hexane (BMH) at 25^o^C for 1 h. The final reaction mixtures were quenched with an equal volume of Laemmli loading buffer (250 mM Tris-HCl, pH 6.8, 10% (W/V) SDS, 0.5% (W/V) BPB, 50% (V/V) glycerol, 5% β-mercaptoethanol, 1 M DTT) and loaded on 12% SDS-PAGE gels. In a large-scale reaction, Flag-tagged ACP4_FkbB_ (0.1 mM) and the mutant S87C-F203L (0.1 mM) were co-incubated in 100 mM sodium phosphate buffer, along with 200 μM BMH for 1 h at 25^o^C. The mixture was buffer exchanged into lysis buffer via a 10-kDa molecular weight cut-off filter and cleaved by the thrombin cleavage capture kit (Novagen, Beijing, China). Then the His-tag of the mixture was removed by Ni^2+^-NTA purification and the desired protein was eluted in the flow-through. The elution was also buffer exchanged into lysis buffer again and subjected to immunoprecipitation with the anti-Flag antibody resin (Qiagen, Valencia, CA, USA). The mixture was eluted with 4 ml of elution buffer (0.1 M glycine (Gly)-HCl, pH 3.5) and concentrated via the cut-off filter. Samples were loaded on 12% SDS-PAGE gels. Lane 1, protein Markers; Lane 2, ACP4_FkbB_ protein; Lane 3, AT4_FkbB_ protein; Lane 4; S89C protein; Lane 5, F203L protein; Lane 6, S89C-F203L protein; Lane 7, the sample of cross-linking of ACP4_FkbB_; Lane 8-11, the sample of cross-linking of AT4_FkbB_(8), S89C(9), F203L(10), S89C-F203L(11) with ACP4_FkbB_ for 1 h; Lane 12, purification of cross-linking complex S87C-F203L-ACP4_FkbB_.

**Reference**:

Vance, S., Tkachenko, O., Thomas, B., Bassuni, M., Hong, H., Nietlispach, D., et al. (2016) Sticky swinging arm dynamics: studies of an acyl carrier protein domain from the mycolactone polyketide synthase. Biochem. J. 473, 1097-1110.

Alekseyev, V.Y., Liu, C.W., Cane, D.E., Puglisi, J.D., Khosla, C. (2007) Solution Structure and Proposed Domain-Domain Recognition Interface of an Acyl Carrier Protein Domain from a Modular Polyketide Synthase. Protein. Sci. 16, 2093-2107.
